# Editorial: Developing therapeutics for antimicrobial resistant pathogens: volume II

**DOI:** 10.3389/fcimb.2025.1581237

**Published:** 2025-03-06

**Authors:** Raja Veerapandian, Parveez Ahamed Abdul Azees, Thiruselvam Viswanathan, Bennett Tochukwu Amaechi, Govindsamy Vediyappan

**Affiliations:** ^1^ Center of Emphasis in Infectious Diseases, Department of Molecular and Translational Medicine, Paul L. Foster School of Medicine, Texas Tech University Health Sciences Center, El Paso, TX, United States; ^2^ Department of Comprehensive Dentistry, School of Dentistry, University of Texas Health Science Center at San Antonio, San Antonio, TX, United States; ^3^ Department of Cellular and Molecular Medicine, Herbert Wertheim College of Medicine, Florida International University, Miami, FL, United States; ^4^ Division of Biology, Kansas State University, Manhattan, KS, United States; ^5^ Diagnostic Medicine and Pathobiology, Kansas State University, Manhattan, KS, United States

**Keywords:** drugs, anti-virulence drugs, anti-biofilm drugs, repurposed drugs, antibiotic-antibody combination therapy, nanotechnology, *in silico* drug design

Antimicrobial resistance (AMR) is one of the most pressing global public health challenges, with bacterial AMR alone directly causing 1.27 million deaths worldwide in 2019 and contributing to 4.95 million deaths, excluding those caused by AMR in viruses, fungi, and parasites (Antimicrobial Resistance, 2022; WHO, 2023). Despite the growing threat of AMR, the development of new therapeutics has stalled, with no clinical candidates close to reaching the market (WHO, 2022). In response to this growing threat, we launched a Research Topic in 2021 titled “*Developing Therapeutics for Antimicrobial-Resistant Pathogens*” (Veerapandian et al., 2022). Building on the success of Volume I, we initiated Volume II in 2024. The latest Research Topic features seven manuscripts presenting diverse perspectives on combating AMR pathogens.

Carbapenem-resistant *Klebsiella pneumoniae* is the major public health problem with high mortality rates (Semet et al., 2024). A comprehensive understanding of its virulence mechanisms and effective therapeutic strategies is critically needed. Tu et al. elucidated the molecular characteristics and pathogenic mechanisms of hypervirulent carbapenem-resistant *Klebsiella pneumoniae* (CR-hvKP) isolated from a human patient sample. Whole-genome sequencing identified three plasmids: Plasmid 1, a pLVPK-like virulence plasmid carrying multiple virulence genes like *rmpA*, *rmpA2*, *iroB*, *iucA*, and *terB*; Plasmid 2, which contains transposable elements such as IS15 and IS26; and Plasmid 3, a resistance plasmid harboring the blaKPC-3 carbapenem resistance gene. Mouse virulence assays with CR-hvKP demonstrated a high mortality rate and a significant increase in proinflammatory cytokine levels, including IL-1β, IL-6, and TNF-α. Interesting article from Aluisio et al. examined the use of cell-free supernatant (CFS) from *Lactobacillus gasseri* against carbapenem-resistant *K. pneumoniae*. Whole genome sequencing of the *L. gasseri* 1A-TV strain revealed two bacteriocin biosynthetic gene clusters (BBGCs) namely BBGC1 which encodes two class IIc bacteriocins, including a gassericin A-like bacteriocin, while BBGC2 harbors the class III bacteriocin helveticin J. Notably, 1A-TV CFS effectively inhibited the growth of all *K. pneumoniae* isolates tested. Nuclear magnetic resonance (NMR) analysis of the *L. gasseri* 1A-TV strain CFS identified and quantified various metabolites involved in carbohydrate and amino acid metabolism, along with organic acids such as ethanol, lactate, acetate, and succinate.

Use of Antimicrobial peptides (AMPs) are one of the promising alternative approaches against AMR pathogens (Xuan et al., 2023; Jeyarajan et al., 2024). Although resistance to AMPs remains relatively low, the potential for resistance development has been reported by multiple mechanism employed by pathogens (Band and Weiss, 2015; Duperthuy, 2020). Hernández-García et al. examined resistance mechanisms associated with Murepavadin, a novel peptidomimetic antibiotic that specifically targets *Pseudomonas aeruginosa* LptD. A genome-wide association study (GWAS) indicated that high resistance to Murepavadin is likely linked to mutations in lpxL1 and lpxL2, resulting in reduced hexa-acylated lipid A, which corresponds to lower inflammatory induction and increased susceptibility to host-derived AMPs.

Combination therapy is an effective approach used recently to treat various bacterial and fungal infections (Basavegowda and Baek, 2022; Bonincontro et al., 2023). Zhao et al. demonstrated that aloe plant extracts containing aloe emodin, emodin, and rhein exhibit strain-specific antibacterial effects against polymyxin-resistant *Acinetobacter baumannii*. Interestingly, combination therapy synergistically restored the sensitivity of resistant *A. baumannii* strains to polymyxins. Moreover, this drug combination showed low cytotoxicity, promoted wound healing, and reduced bacterial burden in mice infected with a polymyxin-resistant *A. baumannii* strain.

Bioactive compounds from the marine ecosystem remain largely unexplored. Metabolites from the marine algae is reported for its antimicrobial activity (Pérez et al., 2016; Makhlof et al., 2024). Sangavi et al. explored the anticariogenic potential of the methanolic extract of *Padina boergesenii* (MEPB). MEPB effectively inhibited *Streptococcus mutans* biofilms without affecting bacterial viability while also impairing key virulence factors such as adherence, acid production, acid tolerance, glucan synthesis, cell surface hydrophobicity, and extracellular DNA (eDNA) production. Its non-toxic nature was confirmed using human buccal epithelial cells. Additionally, GC-MS/MS analysis identified palmitic acid, myristic acid, and stearic acid as the major active constituents of MEPB and the further biofilm inhibition assays with these compounds demonstrated significant antibiofilm activity, with palmitic acid reducing biofilm formation by 85%, myristic acid by 72%, and stearic acid by 83%.

*Candida* species are known to form polymicrobial biofilms with various bacteria, including *Streptococcus* species, *Staphylococcus* species, and *Escherichia coli*, leading to severe clinical outcomes and challenges to treatment (Veerapandian and Vediyappan, 2019; Pohl, 2022). Shaik et al. investigated various phthalimide derivatives, including N-butylphthalimide (NBP), N-methylphthalimide (NMP), N-aminophthalimide (NAP), N-hydroxymethylphthalimide (NHP), N-carbethoxyphthalimide (NCP), and N-(2-butynyl)phthalimide (N2BP), for their antifungal activity against *Candida albicans* and *Candida parapsilosis*. These compounds effectively inhibited biofilm formation, hyphal development, and cell aggregation. Notably, NBP significantly downregulated key hyphal- and biofilm-associated genes (ECE1, HWP1, and UME6) upon treatment. Furthermore, NBP demonstrated broad-spectrum antibiofilm activity by inhibiting biofilm formation of bacterial pathogens such as *Escherichia coli*, *Staphylococcus epidermidis*, *Staphylococcus aureus*, and *Vibrio parahaemolyticus*. Additionally, NBP effectively disrupted polymicrobial biofilms composed of *S. epidermidis* and *C. albicans*. Veerapandian et al. established a mouse model of oropharyngeal candidiasis (OPC) and demonstrated that oral infection with *Candida albicans* can result in its dissemination under immunosuppressed conditions, even in the absence of antibiotics or chemotherapeutic agents. The authors observed alterations in the gut microbiome that favored the proliferation of *Enterococcus* species, *C. albicans*, and potentially other pathogenic bacteria in immunosuppressed mice, which may have exacerbated mucosal damage and facilitated their dissemination.

There are multiple therapeutic strategies available to combat AMR; however, a deeper understanding of its underlying mechanisms is still needed. The recent application of omics technologies has allowed researchers to simultaneously examine multiple layers of molecular activity (Chen et al., 2023). While omics data provide valuable insights into molecular changes following infection, their impact would be significantly greater if these technologies were also applied to therapeutic treatment groups for comparison. This approach would help uncover the molecular mechanisms responsible for the protective effects of therapeutics against diseases (Yow et al., 2022).

Additionally, microbiome studies will play a crucial role in advancing newly developed drugs to the next stage. A key concern when developing new drugs is their potential impact on the host’s normal flora (Patangia et al., 2022). Integrating microbiome analysis with assessments of a drug’s antimicrobial efficacy would provide a more comprehensive understanding of its overall effects.

In summary, we believe that the research articles featured in this Research Topic enhance our understanding of therapeutics for combating antimicrobial-resistant (AMR) pathogens and suggest future directions for addressing AMR challenges.

**Figure 1:**
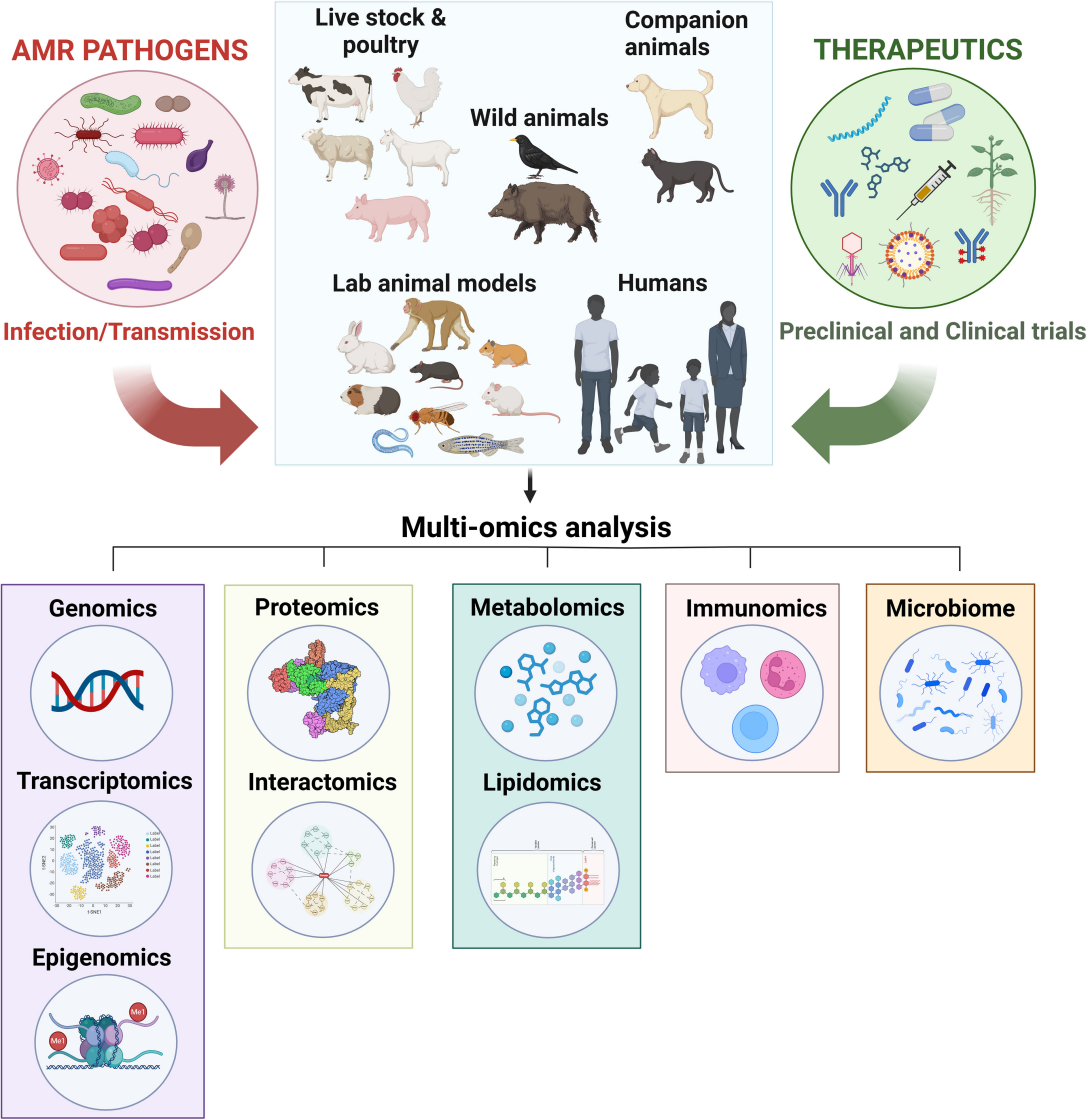
Utilizing omics and microbiome technologies to uncover the molecular mechanisms of therapeutics against AMR. Antimicrobial-resistant (AMR) pathogens, including bacteria, fungi, viruses, and parasites, can naturally infect humans and various animals, such as livestock (e.g., chickens, cattle, goats, sheep, and swine), companion animals (e.g., dogs and cats), and wildlife (e.g., birds and wild boars), leading to disease. Additionally, laboratory animals, including non-human primates, mice, rats, hamsters, guinea pigs, rabbits, zebrafish, *Caenorhabditis elegans*, and *Drosophila melanogaster*, are intentionally infected for research purposes. To combat these AMR pathogens, various therapeutic strategies are employed for treating naturally infected humans and animals, as well as for research on artificially infected laboratory animals. These strategies include plant-derived antimicrobials, antimicrobial peptides, nanomaterials, phage therapy, small compound libraries, drug repurposing, vaccines, antibody, and antibiotic-antibody conjugates. Multi-omics approaches, such as genomics (including transcriptomics and epigenomics), proteomics (including interactomics), metabolomics (including lipidomics), immunomics, and microbiome analysis, can be utilized to elucidate the mechanisms of these therapeutics, accelerating the discovery of effective treatment options.
